# Effect of modified compound calcium phosphate cement on the differentiation and osteogenesis of bone mesenchymal stem cells

**DOI:** 10.1186/s13018-017-0598-8

**Published:** 2017-06-29

**Authors:** Jican Zeng, Jiazhong Lin, Guanfeng Yao, Kangmei Kong, Xinjia Wang

**Affiliations:** 1 Department of Spine Surgery, The Second Affiliated Hospital to Shantou University Medical College, The DongXia North Road, Shantou, 515041 Guangdong China; 2 The Traumatology & Orthopedics Department, The People’s Hospital Affiliated to Fujian University of Traditional Chinese Medicine, Fuzhou, China

**Keywords:** Bone cement, Differentiation induction, Bone morphogenetic protein

## Abstract

**Background:**

The aim of this study is to evaluate the effect of self-invented compound calcium phosphate cement upon the proliferation and osteogenesis of bone mesenchymal stem cells (BMSCs).

**Methods:**

Four groups including traditional calcium phosphate cement, modified calcium phosphate cement, modified calcium phosphate cement plus bone morphogenetic protein (BMP), and control groups were established. The cell proliferation curve was delineated by MTT. The activity of BMSCs to synthesize alkaline phosphatase (AKP) was evaluated. The growth and invasion of BMSCs were observed. The expression levels of aggrecan, collagen I, collagen II, AKP, and OSX messenger RNA (mRNA) were measured by using RT-PCR.

**Results:**

Compared with other groups, the BMSCs in the modified calcium phosphate cement group presented with loose microstructure and the BMSCs closely attached to the vector margin. At 7 days after co-culture, the expression of AKP in the modified calcium phosphate cement plus BMP group was significantly upregulated compared with those in other groups. In the modified calcium phosphate cement group, the BMSCs properly proliferated on the surface of bone cement and invaded into the cement space. At 10 days, the expression levels of aggrecan, collagen I, collagen II, AKP, and OSX mRNA in the modified calcium phosphate cement and modified calcium phosphate cement plus BMP groups were significantly upregulated than those in other groups.

**Conclusions:**

Modified compound calcium phosphate cement possesses excellent biocompatibility and osteogenic induction ability. Loose microstructure and large pore size create a favorable environment for BMSCs proliferation and vascular invasion, as an ideal vector for releasing BMP cytokines to mediate the differentiation and osteogenesis of BMSCs.

## Background

Calcium phosphate cement is a bioactive and biodegradable grafting material in the form of powder and liquid, which, when mixed, sets as primarily hydroxyapatite, occasionally mingled with unreacted particles and other phases. This material has been extensively investigated due to its excellent biomechanical property, potential resorbability, molding capability, and convenient manipulation. Because calcium phosphate cement can potentially be replaced with bone after a period of time, it could retain the short-term biological advantages of hydroxyapatite without the long-term disadvantages. Although little is known about this material in clinical practice, in vivo and in vitro studies have demonstrated that calcium phosphate cement serves as a promising material for grafting applications [1–3]. In our preliminary study, we successfully self-invented a modified absorbable compound bone cement by mixing allogeneic bone powder, β-tricalcium phosphate (β-TCP), and bone powder plus calcium phosphate cement (BCPC) at a fixed proportion [1].

Subsequently, we applied this novel bone cement in animal studies. The experimental results have demonstrated that the self-designed BCPC can induce osteogenesis as bone graft, which possesses excellent molding capability and mechanical property. In addition, it rarely generates the heat energy during the process of solidification. The bone graft properly matches with surrounding bone defects, which reduces the risk of the migration and dislocation of bone fragment. Consequently, this material is of potential significance for the repairing of bone defects and severe comminuted bone fracture in clinical settings. Prior to clinical application, the mechanism underlying the effect of BCPC upon inducing the differentiation and proliferation of bone mesenchymal stem cells (BMSCs) should be elucidated. In this in vitro experiment, BMSCs were isolated from the rabbit models and co-cultured with different types of bone grafting materials to evaluate the effect and clarify the underlying mechanism of inducing proliferation and osteogenesis of BMSCs, aiming to provide fundamental evidence for clinical application of this novel material.

## Methods

### Materials and reagents

Conventional calcium phosphate cement (Shanghai Rebone Biomaterials Co., Ltd., Shanghai, China), β-tricalcium phosphate (β-TCP), hyperpure hydroxyapatite powder (HA) (Sigma, Germany), calcium monohydrogen phosphate and calcium carbonate (Xilong Scientific, Shantou, China), and alginic acid sodium (Alfa Aesar, USA). DMEM culture medium (Hyclone, USA), fetal bovine serum (Sigma, Germany), alkaline phosphatase (AKP) kit (Nanjing Jiancheng Bioengineering Institute, China), RNA extraction kit (BIO BASIC INC., USA), DNA synthesis kit and SYBR Green qPCR kit (Fermentas, USA), and BMP2 (Bone Bank of the Forth Military Medical University, China).

### Methods

#### Preparation of allogeneic bone powder from rabbit models

Fresh cortical bone was collected from the rabbits, and soft tissues were eliminated, cut into pieces with a diameter <1 cm, washed, dehydrated in absolute alcohol for 2 h, soaked in diethyl ether for 1 h, air dried, and stored at −80 °C. According to the Urist technique, the bone particles were crushed into bone powder, sieved through 60–80 mesh into 200–300-μm bone powder, disinfected by radiation exposure, and restored at −80 °C for subsequent use.

#### Preparation of novel compound calcium phosphate cement

The β-TCP, calcium monohydrogen phosphate, calcium carbonate, and hydroxyapatite powder were mingled at a ratio of 6.0:3.0:0.5:0.5 and then mixed with allogeneic bone powder at a ratio of 1.0:0.4, supplemented with 0.25 M Na_2_HPO_4_/NaH_2_PO_4_ buffer solution containing 0.8% alginic acid sodium (pH = 7.4), preserved at 37 °C and 100% humidity for 72 h to create solid blocks of 5 × 5 × 2 mm in size, washed by distilled water, disinfected twice by ultrasonography for 20 min, and air dried, and BCPC blocks were obtained. Bone morphogenetic protein (BMP) (40 mg/g) was mixed with calcium phosphate cement to make BMP plus BCPC blocks according to the methods above.

#### Co-culture of rabbit BMSCs and calcium phosphate cement

Tibial bone marrow was obtained from New Zealand white rabbits and cultured in fresh DMEM culture medium containing 10% fetal bovine serum; the BMSCs were digested using 0.25% trypsin solution at a cell confluence of 90%, passaged at a ratio of 1:3, and the quantity of BMSCs was counted by using trypan blue staining after the second cell passage. Calcium phosphate cement blocks were placed in 24-well plate, supplemented with complete culture solution, and pre-moistured for 1 h; the culture solution was abandoned, the BMSCs were inoculated into the vector at a concentration of 2 × 10^5^ cells/well and cultured at 37 °C and 5% CO_2_; and the culture solution was changed daily. No vector was used in the control group.

#### BMSCs morphology

After cell inoculation, the morphology and growth of BMSCs were observed under inverted microscope. At 6 h, 1-, 3-, and 6 days after cell inoculation, two vectors were collected from the traditional calcium phosphate cement, modified calcium phosphate cement, and modified calcium phosphate cement plus BMP groups; rinsed by phosphate buffer; fixed in 20 g/L glutaraldehyde; dehydrated in gradient ethanol; air dried; and prepared for observation under scanning electron microscope.

#### BMSC proliferation characteristics after surface adhesion

At 24 h after cell inoculation, the BMSCs adhering to both the wall and vector from 5 wells (24-well plate) were digested by trypsin solution. The cell quantity in each well was counted and repeated at 1–7 days after co-culture with BMSCs. The cell growth curve was delineated.

#### Detection of the BMSCs’ ability to secrete AKP

At 1, 3, 5, and 7 days after BMSC inoculation, the BMSCs from 5 wells (24-well plate) were digested by 100 μL Triton-100 solution containing albuminolysis inhibitor and preserved at 4 °C overnight. According to the AKP detection kit instructions, the absorbance value was measured at a wavelength of 410 nm. The cell counting was repeatedly performed at 1, 3, 5, and 7 days after BMSC inoculation. The mean absorbance value was calculated from the values of 1000 cells.

#### Real-time PCR

At 10 days after co-culture, an equivalent quantity of BMSCs was collected from three wells in each group. Total RNA extraction was carried out. RT-PCR was performed to quantitatively measure the expression levels of aggrecan, collagen I, collagen II, AKP, and OSX; the GAPDH was used as internal control. The difference in the mRNA expression was statistically compared using 2^-ΔΔCT^ method [2].

#### Statistical analysis

SPSS 19.0 statistical software was utilized for data analysis (SPSS Inc., Chicago, USA). All data were expressed as mean ± standard deviation (SE). Group comparison was performed by using *t* test. A *P* value of less than 0.05 was considered as statistically significant.

## Results

### Cell morphology and proliferation characteristics of BMSCs after isolation and passage

**Fig. 1:**
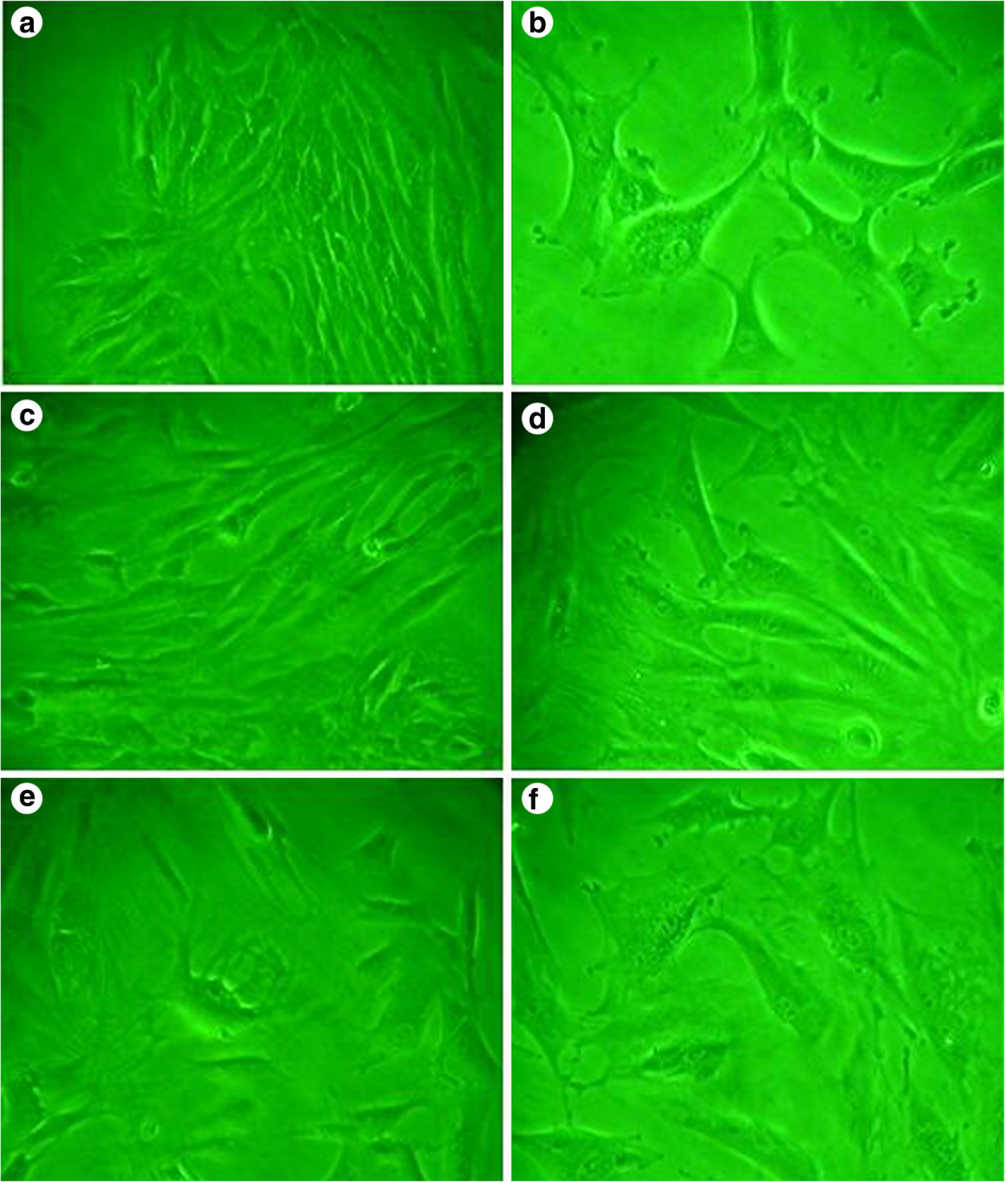
Cell proliferation and morphological changes after BMC-calcium phosphate co-culture (×130). **a** Primary culture cells. **b** The second-generation cells. **c** Cell morphology in the control group at 6 days. **d** Cell morphology in the traditional calcium phosphate group at 6 days. **e** Cell morphology in the BCPC group at 6 days. **f** Cell morphology in the BMP plus BCPC group at 6 days

Primary culture: the culture solution was half exchanged at 24 h and fully exchanged at 72 h. The non-adherent cells were eliminated, which were in long or shuttle shape, and gathered in mass (Fig. 1a). The incubation period of cell proliferation was initiated at 4 to 7 days after primary culture. The mitosis became active at 8 days, the cell cloning was enlarged in exponential growth phase at 9 to 14 days, and the cells completely overspread the bottom of the culture flask at 15 days. Cell passage: during cell passage, the cells proliferated at a faster speed, attached to the wall at 4 h, and evenly overspread the bottom of culture flask at 7 h. No significant change was observed in cell morphology between primary culture and cell passage (Fig. 1b).

### Cell differentiation and proliferation after co-culture of BMSCs and bone cement

**Fig. 2:**
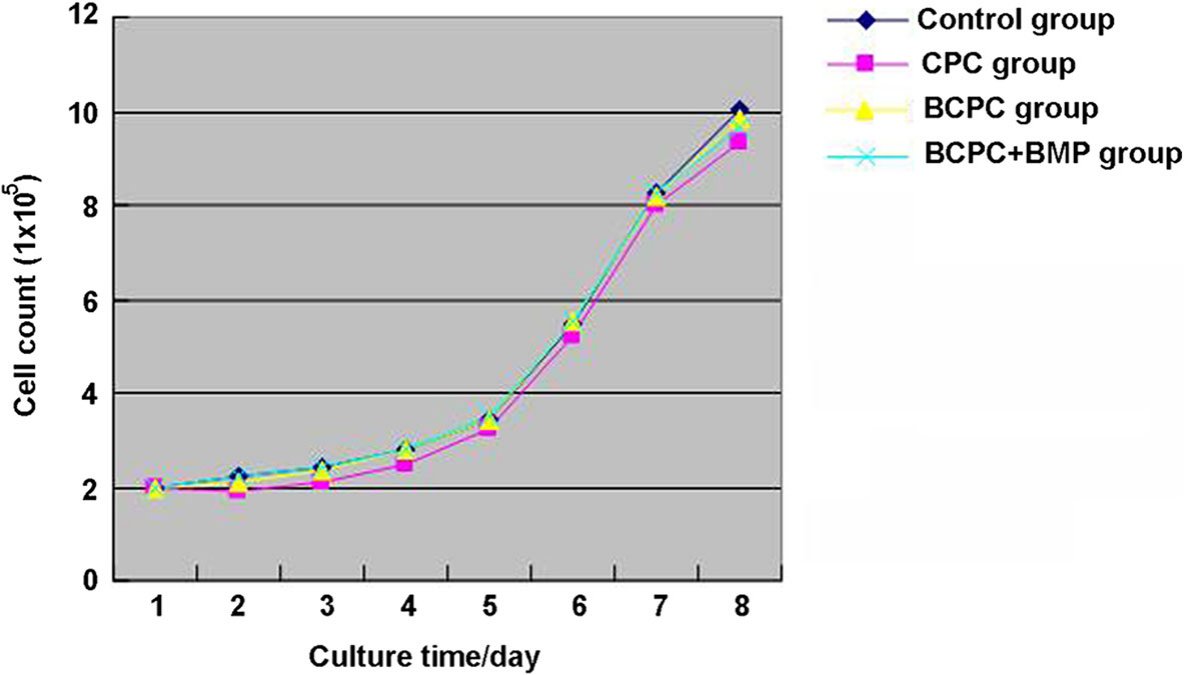
Cell proliferation curve after BMC-calcium phosphate co-culture

After co-culture, the cells closely adhered to the vector margin. Cellular neurite and pleomorphic cells were observed in the BCPC and BMP plus BCPC groups, whereas shuttle-shaped cells were seen in the traditional calcium phosphate cement and control groups, as illustrated in Fig. 1c, d. After cell co-culture, the cells maintained normal cellular division and proliferation. No statistical significance was noted among different groups (all *P* > 0.05), as illustrated in Fig. 2.

### Cell morphology and ultrastructure

**Fig. 3:**
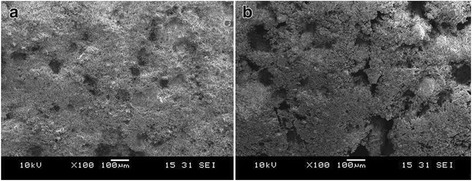
Morphology of bone cement surface under scanning electron microscope (×100). **a** Traditional calcium phosphate cement group. **b** BCPC group

**Fig. 4:**
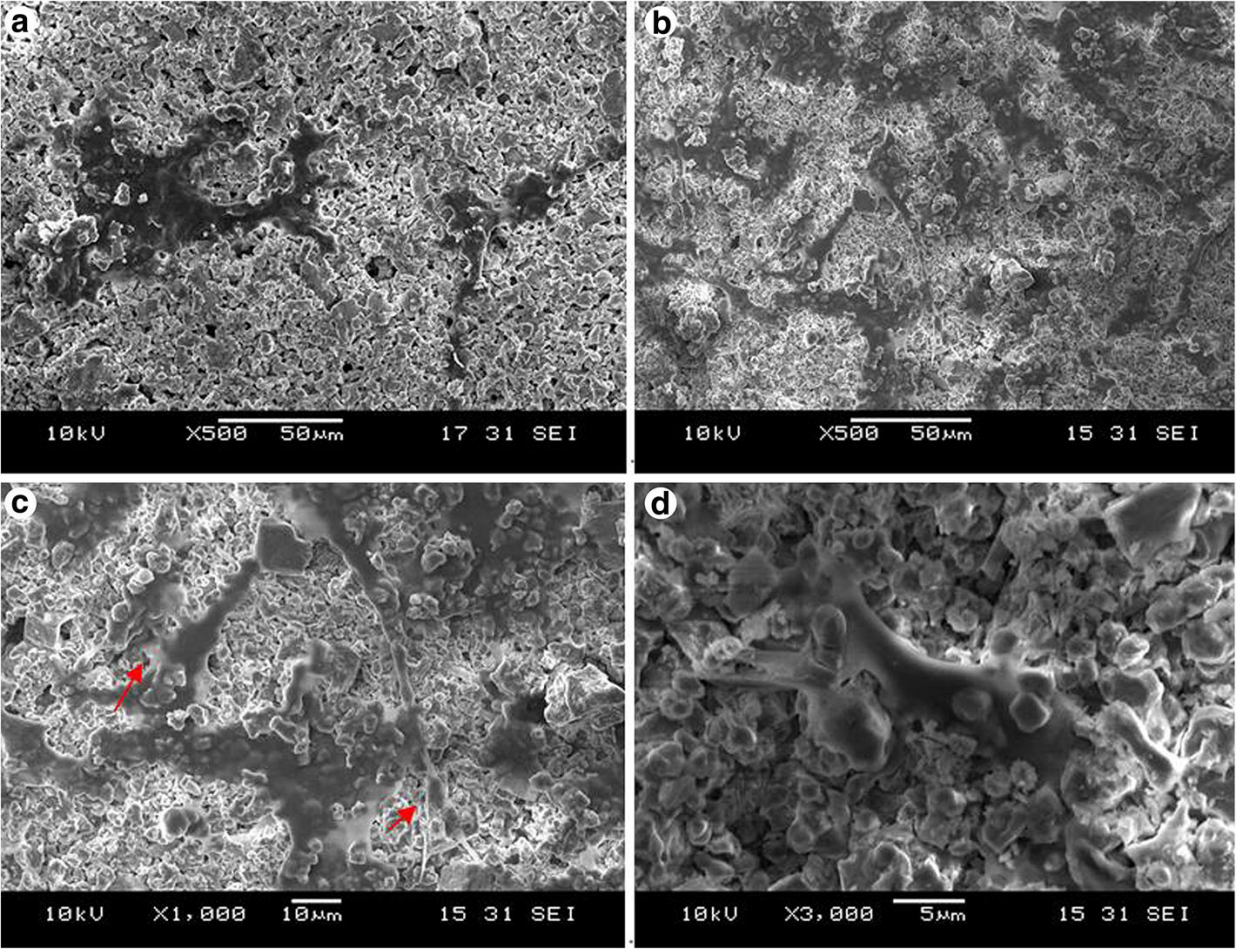
Observation of growth status after BMC-BCPC co-culture at 5 day under scanning electron microscope. **a** At 1 day, the cell quantity was small, which were scattered on the bone cement surface (×500). **b** At 5 days, the cell quantity was significantly increased and gathered in mass on the surface (×500). **c**, **d** The cell pseudopod invaded into the micropore (**c**, ×1000), and the cells grew into the material gap (**d**, ×3000)

Compared with traditional calcium phosphate cement, the modified BCPC had loose structure and large pore size from 200 to 300 μm, as illustrated in Fig. 3. The BMSCs proliferated on the BCPC, outgrew pseudopod, and invaded into the micropore. At 6 days, the cell quantity was significantly increased and gathered in mass surrounding the micropore. A slight quantity of BMSCs extended into the micropore in a shuttle or polygonal shape. In the traditional calcium phosphate cement group, the superficial cell quantity was low and the cells merely proliferated on the BCPC surface. No evident disparity was observed in cell morphology and distribution, as illustrated Fig. 4.

### Detection of AKP activity after cell co-culture

Along with the prolongation of incubation time, the levels of AKP in the traditional calcium phosphate cement and control groups were slightly increased with no statistical significance (both *P* > 0.05). At 7 days after cell co-culture, the AKP content in the BCPC group was significantly higher compared with those in the traditional calcium phosphate cement and control groups (both *P* < 0.05). At 5 and 7 days after cell co-culture, the level of AKP in the BMP plus BCPC group was significantly higher compared with those in other groups (all *P* < 0.05), as illustrated in Table 1.

**Table 1:** Changes in AKP activity after cell co-culture (*x* ± *s*, *n* = 5)

Group	Culture time (d)			
	1	3	5	7
TCP	0.00614 ± 0.00038	0.00609 ± 0.00034	0.00633 ± 0.00029	0.00679 ± 0.00041
BCPC	0.00598 ± 0.00026	0.00617 ± 0.00031	0.00680 ± 0.00038	0.00787 ± 0.00038
BCPC+BMP	0.00600 ± 0.00037	0.00637 ± 0.00042	0.00754 ± 0.00051	0.00992 ± 0.00037
Control	0.00613 ± 0.00029	0.00631 ± 0.00045	0.00640 ± 0.00036	0.00690 ± 0.00028

### Fluorescent real-time PCR

At 10 days after cell co-culture, the expression levels of aggrecan, collagen I, collagen II, AKP, osteogenic, or chondrogenic genes in the BCPC and BMP plus BCPC groups were significantly upregulated compared with those in the traditional calcium phosphate cement and control groups (all *P* < 0.05). No statistical significance was documented in the expression levels of these parameters between the traditional calcium phosphate cement and control groups (all *P* > 0.05), as illustrated in Table 2.

**Table 2:** Expression levels of osteogenic or chondrogenic genes after cell co-culture (2^-ΔCT^ method)

Target genes	Avg.ΔCt (target gene Ct–GAPDH Ct)	ΔΔCt (avg.ΔCt, target-avg.ΔCt control)	Target gene amount relative to control 2^-ΔΔCt^
Collagen I (a)	10.98 + 0.28	0.00 + 0.28	1.0(0.8–1.2)
Collagen I (b)	11.18 + 0.26	0.20 + 0.38	0.9(0.7–1.1)
Collagen I (c)	10.07 + 0.17	−0.91 + 0.33	1.9(1.5–2.3)
Collagen I (d)	8.22 + 0.25	−2.76 + 0.38	6.8(5.2–8.8)
Collagen II (a)	13.15 + 0.20	0.00 + 0.20	1.0(0.9–1.1)
Collagen II (b)	12.89 + 0.17	−0.24 + 0.26	1.2(1.0–1.4)
Collagen II (c)	11.58 + 0.23	−1.57 + 0.30	3.0(2.4–3.7)
Collagen II (d)	10.64 + 0.18	−2.51 + 0.27	5.7(4.7–6.9)
Aggrecan (a)	10.87 + 0.19	0.00 + 0.19	1.0(0.9–1.1)
Aggrecan (b)	10.34 + 0.30	−0.53 + 0.35	1.4(1.2–1.8)
Aggrecan (c)	9.27 + 0.52	−1.60 + 0.55	3.0(2.1–4.3)
Aggrecan (d)	7.62 + 0.30	−3.25 + 0.35	9.5(7.7–11.7)
AKP (a)	11.57 + 0.40	0.00 + 0.40	1.0(0.8–1.3)
AKP (b)	11.70 + 0.52	0.13 + 0.65	0.9(0.6–1.4)
AKP (c)	9.67 + 0.25	−1.90 + 0.47	3.7(2.7–5.1)
AKP (d)	8.27 + 0.28	−3.30 + 0.49	9.8(7.0–13.8)
OSX (a)	14.24 + 0.26	0.00 + 0.26	1.0(0.8–1.2)
OSX (b)	13.86 + 0.21	−0.38 + 0.33	1.3(1.0–1.6)
OSX (c)	12.53 + 0.24	−1.71 + 0.35	3.3(2.6–4.2)
OSX (d)	11.10 + 0.16	−3.14 + 0.31	8.8(7.1–10.2)

## Discussion

Owing to bone diseases and traumatic events, millions of patients worldwide need to undertake bone grafting operations annually [1]. Bone grafting is the procedure of replacing missing or damaged bones with materials from either the patients (autograft) or donors (allograft). There is an increasing demand for synthetic bone substitutes, which are free from the limitations of bone supply, inconsistency, and disease. Moreover, it is of great potential to utilize these substitutes in conjunction with patients’ own cells or recombinant growth factors to accelerate or improve the quality of bone regeneration, which is known as tissue engineering. A wide range of synthetic materials, including metals, ceramics polymers, and cements, have been proposed and developed as bone substitutes. Among them, calcium phosphate cements have been attracting widespread attention due to their excellent biological behaviors, such as biocompatibility, bioactivity, and osteoconductivity. In addition to their excellent biological behavior, the main advantages of calcium phosphate cements are that they can be injected and have the ability to harden in vivo at body temperature. After mixing of the solid and liquid phases, calcium phosphate cements form a viscous paste, which can be easily manipulated and shaped and injected into a defect area, not only avoiding invasive surgical procedures but also providing intimate adaptation to the surrounding bone even for irregularly shaped cavities, representing a unique advantage over bioceramics, which are difficult to machine and shape. Another important feature of calcium phosphate cements is that they are intrinsically microporous. The micropores are left by extra aqueous solution after hardening of calcium phosphate cements due to intergranular spaces, with pore size in the range of submicro- or micrometers. Such micropores are appropriate for the impregnation of biological fluids into calcium phosphate cements and contribute to the resorption and replacement of calcium phosphate cements by bone tissues. However, due to relatively weak cohesion, calcium phosphate cements are likely to disintegrate upon early contact with blood or biological fluids. Another main challenge encountering calcium phosphate cements is that they possess poor mechanical properties in terms of strength toughness, brittleness, and reliability, limiting their application in clinical settings [3, 4].

To resolve these weaknesses, we modified the composition of calcium phosphate cement and mingled allogeneic bone powder with calcium phosphate cement to create novel BCPC. In our preliminary animal experiment, BCPC was proven to act as an artificial bone graft material. The present investigation was designed to evaluate the osteogenic induction effect of BCPC by mixture and co-culture of rabbit BMSCs and BCPC in vitro. Meantime, traditional calcium phosphate cement was also used as the control material. The experimental outcomes have demonstrated that the modified BCPC has loose structure and large pore size above 100 μm compared with the traditional calcium phosphate cement. Therefore, it creates an excellent microenvironment for cellular and vascular proliferation [5]. The proliferation rate of BMSCs did not significantly differ among four groups, suggesting that BCPC and BMP impose no evident toxicity upon the BMSCs and yield excellent cellular biocompatibility. At 7 days after cell co-culture, the activity of AKP in the BCPC and BMP plus BCPC groups was significantly higher compared with the traditional calcium phosphate cement and control groups. As a pivotal biomarker of osteoblast differentiation, high AKP activity indicates the high differentiation ability from menchymal stem cells to osteoblasts [6]. RT-PCR demonstrated that the expression levels of aggrecan, collagen I, collagen II, AKP, and osteogenic or chondrogenic genes in the BCPC and BMP plus BCPC groups were significantly upregulated compared with those in the traditional calcium phosphate cement and control groups, especially the BMP plus BCPC group, suggesting that BCPC has certain osteogenic induction activity, which is significantly enhanced after supplement with BMP.

Modified Urist method can properly preserve and release BMP cytokines [7, 8]. BMP is capable of inducing cells with osteogenic potential to differentiate into osteoblasts. Co-culture of BMP from natural extract or gene recombination and vector can repair and accelerate the regeneration of bone tissues [9]. In this investigation, we modified and improved the Urist method by mixing allogeneic bone powder with calcium phosphate cement material to create BCPC, which possesses loose pore structure, which contributes to the proliferation and differentiation of target cells [10]. Previous animal studies have demonstrated that calcium phosphate cement has certain osteogenic induction ability [11–15]. In the present experiment, no significant osteogenic induction activity was observed in the traditional calcium phosphate cement and control groups, probably because the calcium phosphate cement absorbs surrounding active cytokines and possesses certain biological activity. It may also result from the different physicochemical properties of different materials. In this experiment, bone powder rather than the mixture of bone particle and calcium phosphate powder was utilized mainly because compared with bone particle, bone powder favors the release of BMP. The diameter of bone particles is relatively large, which induces injuries to crosslinking structure after hydration response of calcium phosphate, thereby affecting the molding of bone cement. Early filling of bone powder functions to support the hydroxyapatite gap following hydration reaction of calcium phosphate cement. Compared with bone particles, bone powder possesses faster degradation and absorption due to smaller diameter, which contributes to subsequent vascular proliferation and invasion. After multiple screening experiments, the optimal ratio of bone powder and calcium phosphate powder is 1:0.4. An insufficient quantity of bone powder affects the biological activity of bone cement. Excessive supplement of bone powder yields bone cement clotting. However, the expression level of collagen III was not quantitatively investigated. In addition, the expression levels of relevant growth factors remain to be elucidated, which contributes to unravel the mechanism underlying the effect of the modified cement. We have supplemented these experiments in our subsequent research.

## Conclusions

Novel compound calcium phosphate cement has proven to possess excellent biocompatibility and osteogenic induction ability. Loose microstructure and large pore size create a favorable environment for BMSCs’ proliferation and vessel invasion. It serves as an ideal vector for releasing BMP cytokines, thereby inducing and mediating the differentiation and osteogenesis of BMSCs.

## Abbreviations

AKP: Alkaline phosphatase
BCPC: Bone powder plus calcium phosphate cement
BMP: Bone morphogenetic protein
BMSCs: Bone mesenchymal stem cells
SE: Standard deviation
β-TCP: β-tricalcium phosphate
